# Supplementary material for: Data modeling analysis of GFRP tubular filled concrete column based on small sample deep meta learning method
Source: PLoS One. 2024 Jul 10;19(7):e0305038. doi: 10.1371/journal.pone.0305038 (PMC11236119; doi:10.1371/journal.pone.0305038)
Supplement: Attachment — Submitted filename: Reviewer Comments to Author.pdf [file pone.0305038.s002.pdf]

Review Comments to Author

Review for “Data modeling analysis of GFRP tubular filled concrete column based on small sample deep meta learning method”

Reviewer Comments:

Data modeling analysis of GFRP tubular filled concrete column based on a small sample deep meta-learning method is investigated in this study. The reviewer suggests it can be reconsidered if the authors can address the comments as follows:

1. Page 1, the manuscript in the title page (Title, Author, Affiliations, Abstract) lacks the line number, which makes it too difficult for the reviewer to locate the possible comments on this study.
2. Page 1, Author lists, Tianyi Deng[1☯], Chengqi Xue[1☯], Gengpei Zhang[1*]. What does the symbol “☯” mean for the authors? Maybe it represents the authors’ contribution equally to this research. In addition, what is this symbol “*” stands for? Corresponding author or other meanings? Considering the E-mail address is provided in the next line. Please clarify. Add the corresponding author before the E-mail address.
3. Page 1, the affiliation “, Yangtze University, Jingzhou City, Hubei Province, China”. The school of affiliation is correct? The reviewer recommends “School of Electronics and Information”. Please confirm this. The postcode is neglected in the affiliation.
4. Page 3, Line 76, Introduction, “using concrete filled GFRP column data as the test object to solve the modeling and analysis problem of small sample data in building structural units”. The abbreviation GFRP is the first time appeared in the manuscript without providing the full name. Please check the whole manuscript for similar issues.
5. The manuscript is entitled “GFRP tubular filled concrete column”. However, the reviewer could not find any information about why GFRP tubular filled concrete columns were chosen for the research object. Please explain the reasons.

6. The illustration of the Figs. 1~4, 7~8 was low resolution with poor quality. The reviewer suggests the authors redraw the flowchart by using Visio software. Special attention should be paid to the font, preferably Times New Roman.
7. Table 1, function “f and F” should be changed into “ f and F ”. Please check all the symbols in the whole manuscript. In the process, (X, Y), y=f(x), f=F(), please use the MathType software for the symbols, Italic Font.
8. Figs. 5 and 6 should be cited because they are not your own tested specimens.
9. Figs. 9~13 is the same style, they could combine in the same figure with sub-figure (a)~(d). Considering your expertise, please change the drawing style from Excel to Origin, Matlab, or Python. Vector graph is highly recommended in scientific graphs. Figs. 14~18 is a similar situation.
10. Page 10, Tables 2 and 3, “ultimate displacement” should be replaced by “Ultimate displacement”, which can consist of “Evaluation criteria” and “Evaluation criteria”.
11. Page 15, Lines 351~352, This method is superior to SVMR, GPR, and RBFNN in solving small sample data modeling of beams/columns. Does the proposed method consider GFRP-reinforced concrete beams? Please clarify.
12. Page 15, Lines 354~357, H/D and D/T should be replaced by “ H/D and D/T ”. Please check similar issues in the whole manuscript.
13. Please add DOI for the references listed.
14. Much literature has been reviewed in the paper, but less critical. The latest research concerning this topic should be addressed and added in the introduction. In other words, you should clearly explain what contribution that has been made by former research and, in particular, what limitation or weakness that exists in each previous research. You should identify the gap in the knowledge from the review of the previous research to justify the significance of YOUR current research topic.
15. Some assumptions are stated in various sections. Justifications should be provided for these assumptions. What is the basic principle of SVR, GPR, RBFNN, and SSDML? Evaluation of how they will affect the results should be made.

16. What are the concrete and GFRP material properties of the tested specimens? How to calculate the ultimate bearing capacity?
17. The authors should clarify the novelty of this manuscript.
18. The background and research needed on GFRP should be demonstrated with more words in the introduction section.
19. The authors should clarify how this method is to be used for GFRP composite structures in civil engineering.
20. The data points in this research are not quite enough. How the authors can guarantee the accuracy of this modeling approach?
21. Page 13, line 308. As shown in Fig 14 Fig 18, the ultimate displacement of the column decreases with the increase of the symmetry ratio. Figs. 14 and 18.
22. The comparison of the data is too common with only qualitative analysis, please rewrite the style with quantitative analysis.

In its current version, the reviewer recommends the document for Major Revision in *PLOS ONE*.
